# Epigenomic insights into CD4^+^ T-cell–mediated pathogenesis in autoimmune diseases

**DOI:** 10.3389/fimmu.2026.1974558

**Published:** 2026-10-27

**Authors:** Manaka Goto, Hiroyuki Teruya, Tomohisa Okamura, Keishi Fujio

**Affiliations:** 1 Department of Functional Genomics and Immunological Diseases, Graduate School of Medicine, The University of Tokyo, Tokyo, Japan; 2 Department of Allergy and Rheumatology, Graduate School of Medicine, The University of Tokyo, Tokyo, Japan

**Keywords:** autoimmunity, epigenetics, multi-omics, single-cell analysis, T cells

## Abstract

T cells are central mediators of autoimmune disease pathogenesis. After developing in the thymus, T cells undergo diverse differentiation programs and acquire specialized effector functions in inflamed tissues. Despite sharing the same genome, T cells adopt diverse phenotypes and functions, highlighting the importance of epigenetic mechanisms in regulating their differentiation and activity; however, these mechanisms remain incompletely understood. Comprehensive epigenetic analyses are therefore essential to elucidate these processes. To date, a wide range of approaches has been applied to T-cell research, including analyses of DNA methylation, histone modifications, chromatin accessibility, transcription factor binding, and three-dimensional chromatin architecture. More recently, rapid advances in single-cell technologies have enabled simultaneous profiling of gene expression and chromatin accessibility in the same cell. In parallel, CRISPR-based approaches have enabled genome-wide functional interrogation in primary human T cells, including analyses of non-coding regulatory regions. Furthermore, epigenomic approaches are now being applied to spatial analyses and are expected to provide deeper insight into the organization of T-cell states in inflamed tissues. In this mini review, we highlight recent studies that have leveraged these cutting-edge technologies and discuss how they are advancing our understanding of the T-cell–mediated pathogenesis of autoimmune diseases.

## 1 Introduction

Epigenetics classically refers to regulatory mechanisms that alter gene activity without changing the underlying DNA sequence, including DNA methylation and histone modifications. More broadly, epigenomic profiling captures regulatory features such as chromatin accessibility, transcription factor occupancy, and three-dimensional chromatin organization. Although most cells of an individual share essentially the same genomic sequence, different immune cell types, such as T and B cells, exhibit characteristic epigenetic states that establish cell type-specific transcriptional programs and, consequently, distinct functional properties. External stimuli, including infection, inflammation, and changes in the metabolic environment, can also reshape chromatin states in concert with transcription factor activation, thereby contributing to transient or persistent alterations in immune-cell function. Because both genetic predisposition and environmental factors contribute to the development and progression of autoimmune diseases, epigenetic regulation represents an important molecular interface through which these signals are translated into cellular phenotypes. Understanding this interface is therefore essential for elucidating the pathogenesis of autoimmune diseases. Moreover, because at least some epigenetic alterations are reversible, epigenetic regulators have attracted considerable interest as therapeutic targets. Epigenetic profiling technologies have also advanced rapidly. Whereas early studies focused primarily on specific genomic regions, genome-wide characterization of the epigenome has become a central approach. The major epigenomic approaches, their biological interpretations, and their key limitations are summarized in **Table 1**. In this mini review, we discuss how advances in epigenomic profiling have refined our understanding of the contribution of CD4^+^ T cells to autoimmune disease pathogenesis (**Figure 1**). Reflecting the relative depth of available epigenomic data, we place particular emphasis on systemic lupus erythematosus (SLE) and rheumatoid arthritis (RA), while also highlighting relevant findings from multiple sclerosis (MS), Sjögren’s disease (SjD), and systemic sclerosis (SSc).

**Table 1:** Comparison of major approaches for investigating epigenetic and chromatin regulation in autoimmune diseases.

Regulatory layer	Representative methods	Resolution	Biological inference	Main limitation
DNA methylation	DNA methylation array/BS-seq	Bulk/single-cell	DNA methylation states at promoters and other regulatory regions	Relationship with transcription is context-dependent
Chromatin accessibility	ATAC-seq	Bulk/single-cell/spatial	Accessible chromatin and candidate regulatory elements	Accessibility does not establish regulatory function or target genes
Histone modifications/TF occupancy	ChIP-seq, CUT&RUN, CUT&Tag	Bulk/single-cell	Chromatin states associated with active or repressed regulatory elements; genomic occupancy of TFs and other chromatin-associated proteins	Histone marks or TF occupancy do not establish regulatory function
3D chromatin structure	Hi-C, Capture-C, HiChIP	Bulk/single-cell	Higher-order chromatin organization and physical contacts between distal regulatory elements and promoters	Physical contact does not prove regulatory effect

The table summarizes the regulatory layer assessed, representative methods, analytical resolution, biological inference enabled, major limitations. BS-seq, bisulfite sequencing; ATAC-seq, assay for transposase-accessible chromatin with sequencing; ChIP-seq, chromatin immunoprecipitation sequencing; CUT&RUN, cleavage under targets and release using nuclease; Hi-C, high-throughput chromosome conformation capture; Capture-C, chromosome conformation capture coupled with targeted sequence capture; HiChIP, Hi-C coupled with chromatin immunoprecipitation; TF, transcription factor.

**Figure 1:**
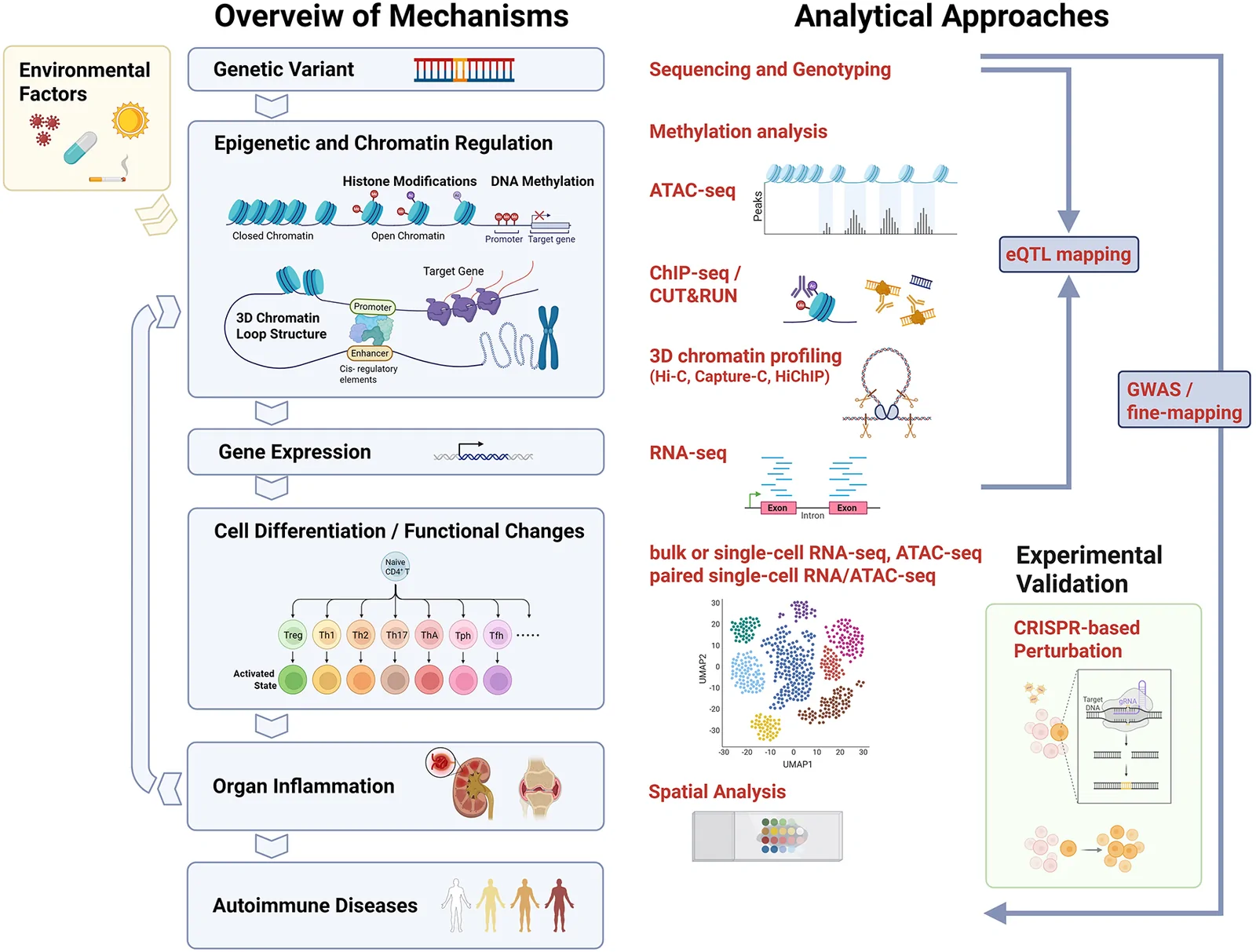
Overview of epigenomic approaches for understanding CD4^+^ T-cell dysregulation in autoimmune diseases. Genetic variants and environmental factors influence multiple layers of epigenetic and chromatin regulation, including DNA methylation, histone modifications, chromatin accessibility, transcription factor occupancy, and three-dimensional chromatin architecture. These changes can influence gene expression and CD4^+^ T-cell differentiation and function, thereby contributing to organ inflammation and autoimmune disease. Conversely, inflammatory microenvironments can reshape epigenetic states in CD4^+^ T cells, providing a mechanism through which disease-associated inflammation may further modify CD4^+^ T-cell phenotypes. Integrated genomic, epigenomic, single-cell, spatial, and CRISPR-based approaches enable the identification of disease-associated regulatory mechanisms and the functional assessment of candidate regulatory elements and genes. ATAC-seq, assay for transposase-accessible chromatin with sequencing; ChIP-seq, chromatin immunoprecipitation sequencing; CUT&RUN, cleavage under targets and release using nuclease; Hi-C, high-throughput chromosome conformation capture; Capture-C, chromosome conformation capture coupled with targeted sequence capture; HiChIP, Hi-C coupled with chromatin immunoprecipitation; CRISPR, clustered regularly interspaced short palindromic repeats; eQTL, expression quantitative trait locus; GWAS, genome-wide association study. Created with BioRender.com.

## 2 Epigenetic dysregulation of T cells in autoimmune diseases

Dysregulated epigenetic mechanisms contribute to T-cell dysfunction in autoimmune diseases. Historically, hydralazine and procainamide, two drugs associated with drug-induced lupus, were shown to inhibit DNA methylation in cloned T cells and induce autoreactivity (1). Reduced DNA methylation capacity has also been reported in T cells derived from patients with SLE or RA (2). Subsequent genome-wide epigenomic studies have demonstrated disease-associated DNA methylation alterations in CD4^+^ T cells or specific CD4^+^ T-cell subsets across autoimmune diseases, including SLE, RA, SjD, and SSc (3–6). While DNA methylation provides one layer of epigenetic regulation, histone modifications offer complementary information about regulatory activity. Histone H3 lysine 27 acetylation (H3K27ac) marks active enhancers and promoters (7), whereas histone H3 lysine 4 trimethylation (H3K4me3) is enriched around active transcription start sites (8). Consistent with the role of H3K27ac as a marker of active regulatory elements, the landscape of H3K27ac-marked enhancers in CD4^+^ T cells undergoes disease-specific remodeling across several autoimmune diseases, including SLE (9).

SLE is a systemic autoimmune disease characterized by loss of immunological self-tolerance and inflammation affecting multiple organs. Enhanced type I interferon signaling and autoantibody production are prominent features of the disease (10). In SLE, hypomethylation of genomic regions surrounding type I interferon-responsive genes has been consistently observed in several immune-cell populations, including CD4^+^ T cells. In naïve CD4^+^ T cells, hypomethylation at interferon-responsive loci is consistent with a poised transcriptional state, which may facilitate rapid induction of these genes in response to inflammatory signals (3, 11, 12). Furthermore, a study comparing SLE-affected and unaffected individuals within monozygotic twin pairs identified disease-associated DNA methylation differences in the peripheral blood leukocytes of the affected twins. These findings demonstrate disease-associated epigenetic differences independent of germline sequence variation, although such comparisons do not eliminate confounding by differences in cellular composition, environmental exposures, disease activity, and treatment (13). Purified populations and single-cell profiling can help address cellular-composition confounding.

Although cross-sectional studies establish robust disease-associated epigenetic alterations, they do not distinguish whether epigenomic alterations precede disease onset and promote pathogenesis or arise as a consequence of chronic inflammation. Longitudinal studies in RA have begun to address this question. RA is an autoimmune disease characterized by persistent inflammation of the synovial joints, and anticitrullinated protein antibodies (ACPAs) can be present years before the onset of arthritis (14). Recently, longitudinal multi-omics profiling of CD4^+^ T cells from ACPA-positive individuals without clinically apparent inflammatory arthritis identified epigenetic changes in naïve CD4^+^ T cells, including increased chromatin accessibility at the *CXCR5* and *IL21* loci (15). *CXCR5* encodes a chemokine receptor that promotes migration toward B-cell follicles in secondary lymphoid tissues (16), whereas IL-21 is a cytokine that supports B-cell maturation and antibody production. These alterations may predispose naïve CD4^+^ T cells to differentiate into effector T cells with B-cell-helper functions. Progression to clinical RA was additionally associated with expansion of memory CD4^+^ T-cell states characterized by activation and B-cell-helper signatures. If longitudinal epigenomic studies can identify changes that precede disease onset or clinical relapse, they could improve understanding of autoimmune pathogenesis while also enabling disease prediction, patient stratification, and optimization of therapeutic timing.

## 3 Cell-state-specific regulatory effects of autoimmune risk variants in CD4^+^ T cells

Although longitudinal epigenomic studies can identify changes preceding disease onset, genetic studies provide a complementary approach for prioritizing potentially causal regulatory mechanisms. Numerous genome-wide association studies (GWAS) have identified susceptibility loci for autoimmune diseases such as SLE and RA. However, GWAS signals alone often do not uniquely identify causal variants, partly because each association signal may contain multiple correlated variants (17). Farh et al. fine-mapped GWAS loci across 21 autoimmune diseases to identify candidate causal single-nucleotide polymorphisms (SNPs) and estimated that approximately 90% of these putative causal variants were located in non-coding regions and that approximately 60% overlapped enhancers active in immune cells (17). Analysis of cell type-specific H3K4me3 profiles further demonstrated that RA-associated variants were enriched in active chromatin regions in CD4^+^ T cells, particularly regulatory T (Treg) cells (18). CpG-demethylated regions specific to naïve Treg cells have also been associated with Treg-specific gene expression and chromatin states. SNPs associated with multiple autoimmune diseases were preferentially enriched within demethylated regions specific to naïve Treg cells, whereas this enrichment was not observed within activation-induced demethylated regions shared by Treg and conventional T cells (19).

The development of single-cell technologies has enabled disease-associated regulatory features to be resolved at the level of individual T-cell populations. For example, bulk assay for transposase-accessible chromatin with sequencing (ATAC-seq) of peripheral blood CD4^+^ T cells from patients with SLE revealed disease-severity-associated chromatin changes, including a T-cell exhaustion-related signature. Consistent with these findings, single-cell RNA-seq identified a CCR7^low^CD74^high^ Treg population characterized by a type I interferon response and exhaustion-like transcriptional programs (20). Simultaneous single-nucleus ATAC-seq and RNA-seq analysis of naïve CD4^+^ T cells from patients with SLE further linked disease-associated chromatin accessibility changes to transcriptional alterations and showed that these changes extended across multiple naïve T-cell subpopulations (21).

Importantly, disease-associated regulatory elements are not necessarily constitutively active. Epigenomic profiling under defined stimulation conditions has shown that autoimmune disease risk is enriched not only within accessible chromatin in resting immune cells but also within regulatory elements that emerge or become more active following stimulation (22). Soskic et al. examined the activity of regulatory regions containing immune disease-associated variants across the time course of T-cell activation. For many diseases, variant enrichment was stronger during early activation than later activation of memory CD4^+^ T cells (23).

Subsequent single-cell expression quantitative trait locus (eQTL) studies, which identify genetic variants statistically associated with gene expression variations, demonstrated that the effects of genetic variants on gene expression can likewise depend on stimulation and the cellular state. By linking genetic variation to gene expression, eQTL mapping complements epigenomic analyses in identifying candidate target genes of regulatory variants. Soskic et al. profiled CD4^+^ T cells from 119 healthy individuals at baseline and several time points after stimulation. A subset of eQTL effects changed dynamically during the activation of naïve and memory CD4^+^ T cells (24). Complementing these findings, Nathan et al. analyzed unstimulated memory T cells along continuous functional states and showed that eQTL effects varied according to cellular properties such as cytotoxicity and regulatory capacity. Autoimmune disease-associated variants were enriched among these cell-state-dependent eQTLs (25).

Transcriptional activity at enhancers provides an additional layer of information beyond chromatin accessibility. Oguchi et al. identified enhancers with bidirectional enhancer RNA transcription, a feature associated with active enhancer function, using single-cell RNA sequencing. Immune disease-associated genetic variants were enriched more strongly in these transcriptionally active, cell-state-specific enhancers than in open chromatin regions defined by accessibility alone (26). Collectively, these studies indicate that defining the cellular context in which disease-associated variants exert regulatory effects requires consideration not only of discrete T-cell subsets but also of stimulus identity, time after stimulation, and continuous functional states. Nevertheless, overlap between disease-associated variants and epigenomic features, or colocalization with an eQTL signal, is insufficient to establish the direct target gene of a distal regulatory element or its causal effect on T-cell function. Linking non-coding variants to disease mechanisms therefore requires three-dimensional genome approaches that identify enhancer–promoter interactions, followed by experimental perturbation of the candidate regulatory elements or target genes.

## 4 Distal gene regulation through three-dimensional chromatin contact

Regulatory regions containing non-coding variants have traditionally been assumed to regulate the nearest gene along the linear genome. However, advances in chromosome conformation capture technologies, including Hi-C, have demonstrated that distal regulatory elements such as enhancers can interact with promoters located tens of kilobases to over 1 Mb away within the three-dimensional organization of the nucleus. Hi-C is used to provide genome-wide maps of chromatin contacts via sequencing of DNA fragments that are spatially proximal in the nucleus. Using promoter capture Hi-C, which enriches promoter-associated chromatin contacts, in 17 primary human hematopoietic cell types, Javierre et al. demonstrated that regions containing non-coding disease-associated variants can interact with distal promoters. Many of these interactions were cell-type specific (27). Promoter capture Hi-C of resting and activated CD4^+^ T cells, integrated with fine-mapped GWAS signals for five autoimmune diseases including RA and SLE, prioritized 245 protein-coding genes whose promoters interacted with genomic regions containing disease-associated signals (28). Of these genes, 108 were linked through activation-sensitive chromatin interactions. The median distance between the peak GWAS signal and the prioritized gene was 153 kb, illustrating how nearest-gene assignment can overlook distal candidate target genes and activation-state-specific regulatory relationships.

Many enhancers containing autoimmune disease-associated variants were found to interact with genes other than the nearest gene in the linear genome in naïve CD4^+^ T cells, T helper 17 cells, and Treg cells, as demonstrated by H3K27ac HiChIP, which detects chromatin contacts involving H3K27ac-marked regulatory regions (29). The Activity-by-Contact model provides a complementary computational framework for predicting enhancer–gene relationships by integrating enhancer activity with enhancer–promoter contact frequency (30). More recently, integration of promoter-focused Capture-C, Hi-C, ATAC-seq, and RNA-seq data across 57 human cell types enabled the generation of a variant-to-gene map for 16 autoimmune diseases and traits, including SLE (30). This analysis predicted the cell types in which variants were likely to exert their effects and the genes they potentially regulate. Although many candidate variants were disease specific, a substantial proportion of their predicted target genes were shared across autoimmune diseases and cell types, suggesting that distinct genetic risk variants can converge on common biological networks (31).

Despite these advances, comprehensive studies of three-dimensional genome organization in T cells from patients with autoimmune diseases remain limited. Zhao et al. integrated Hi-C and RNA-seq analyses of CD4^+^ T cells from patients with SLE and healthy controls and identified chromatin-loop alterations associated with disease activity (32). Motif and regulatory analyses implicated a role for SPI1 in regulating the interferon-response gene *DDX60L* at a locus with altered chromatin architecture. These findings illustrate the potential of three-dimensional genome profiling to link disease-associated regulatory regions with distal target genes. However, physical contact alone does not establish direct transcriptional regulation, underscoring the need for functional validation via perturbation of the candidate regulatory element or target gene.

## 5 Establishing functional causality

Integration of epigenomic, eQTL, and three-dimensional genome analyses has enabled the identification of numerous candidate regulatory elements and target genes through which disease-associated variants may act. However, these approaches primarily identify statistical associations or physical proximity. Direct perturbation is required to determine whether a candidate regulatory element actually regulates gene expression or T-cell function. Beyond CRISPR–Cas9-mediated gene knockout, CRISPR interference (CRISPRi) and CRISPR activation (CRISPRa) enable systematic interrogation of promoters, enhancers, and protein-coding genes by repressing or activating transcription without introducing double-strand breaks in the target sequence. Shifrut et al. developed single-guide RNA lentiviral infection with Cas9 protein electroporation (SLICE) to conduct genome-wide CRISPR loss-of-function screening in primary human T cells (33). Using this approach, they identified both established and previously unrecognized regulators of T-cell proliferation following T-cell receptor stimulation. For selected perturbations, downstream transcriptional programs were further characterized by single-cell RNA sequencing. Related Perturb-seq approaches systematically link individual CRISPR perturbations to single-cell transcriptomic readouts, allowing analysis of downstream gene-expression programs and cellular states. Alda-Catalinas et al. used the Perturb-seq approach to perturb 45 distal regulatory elements and 35 transcription start sites and quantified the resulting transcriptional responses across approximately 250,000 primary CD4^+^ T cells (34). The results provided functional support for multiple regulatory element–gene connections.

Functional perturbation has also been applied specifically to autoimmune disease-associated regulatory elements. Daga et al. integrated H3K27ac profiles and transcriptomes from CD4^+^ T cells obtained from patients with several autoimmune diseases and healthy individuals to infer disease-specific, enhancer-based gene regulatory networks (9). Chromatin-capture data supported candidate relationships between enhancers containing disease-associated SNPs and their putative target genes, and selected relationships were functionally tested using CRISPRi in primary human CD4^+^ T cells. The study suggested that disease-specific enhancer remodeling occurs in addition to inflammatory transcriptional programs shared among autoimmune diseases. More recently, Ho et al. functionally interrogated more than 18,000 autoimmune disease-associated variants using massively parallel reporter assays together with bulk and single-cell CRISPRi in primary human CD4^+^ T cells. This large-scale analysis provided functional evidence linking selected variant-containing regulatory elements to target-gene expression and T-cell proliferation (35).

In Treg cells from patients with multiple sclerosis, an immune-mediated demyelinating disease of the central nervous system, large-scale alterations in overall chromatin accessibility were less prominent than changes in transcription factor footprints within pre-existing accessible regions (36). These footprints were consistent with increased occupancy by AP-1 and interferon regulatory factor family transcription factors. CRISPRa and CRISPRi perturbation of a distal regulatory element showed that it preferentially regulates PRDM1-S, the short isoform of PRDM1. PRDM1-S induces SGK1, thereby destabilizing FOXP3 and impairing Treg function. These findings indicate that disease-associated epigenomic dysregulation cannot be understood solely by examining chromatin accessibility; it also requires analysis of transcription factor occupancy and the isoform-specific effects of regulatory activity. Law et al. identified a regulatory mechanism underlying the differentiation of pathogenic CXCL13-producing peripheral helper T and follicular helper T cells, which are expanded in SLE, by integrating CRISPR screening, ATAC-seq, and CUT&RUN analyses in human CD4^+^ T cells (37). CUT&RUN is a high-resolution alternative to chromatin immunoprecipitation sequencing (ChIP-seq) for genome-wide mapping of chromatin-associated proteins, including transcription factors. The results identified AHR as a suppressor of the CXCL13-producing T-cell phenotype. Mechanistically, AHR and JUN favored an alternative differentiation state associated with IL-22 production, whereas type I interferon disrupted this regulatory program and shifted activated CD4^+^ T cells toward CXCL13 expression. This work functionally linked the inflammatory cytokine environment in SLE with transcription factor occupancy, epigenomic state, and pathogenic T-cell differentiation.

Advances in CRISPR technologies have thus expanded functional analyses from genome-wide gene screens to studies focused on disease-associated variants, individual regulatory elements, and inflammatory environments. However, the regulatory effects observed using CRISPRi or CRISPRa depend on the T-cell subset, stimulation conditions, culture environment, and time point examined. Determining whether the same regulatory relationships operate in inflammatory tissues from patients is therefore essential for mechanistic interpretation and therapeutic translation. Technologies that preserve tissue context may help address this limitation. In parallel with single-cell Hi-C approaches (38), spatial ATAC-seq and spatial CUT&Tag have been developed to profile chromatin accessibility and histone modifications in tissue sections (39–41), although their application to CD4^+^ T cells from patients with autoimmune diseases remains limited. Spatial multi-omic approaches have also enabled epigenomic and transcriptomic information to be integrated within the same tissue context. These technologies may permit direct investigation of how local cytokine exposure and interactions with neighboring immune and stromal cells are associated with pathogenic epigenomic states in tissue-infiltrating T cells—relationships that cannot be fully captured by analyses of peripheral blood. Nevertheless, current spatial epigenomic methods remain limited by spatial resolution, detection sensitivity, tissue requirements, and cost. Their application to tissues from patients with autoimmune diseases therefore represents an important area for future investigation.

## 6 Discussion

Advances in epigenomic technologies have established that T-cell abnormalities in autoimmune diseases involve multiple layers of epigenetic and chromatin dysregulation, including DNA methylation, histone modifications, chromatin accessibility, and three-dimensional genome organization. In particular, many autoimmune disease-associated variants are located in non-coding regulatory regions, and their regulatory effects may be restricted to, or become more pronounced in, specific T-cell subsets and activation states. However, cross-sectional patient studies cannot readily distinguish epigenomic alterations that contribute to disease onset or exacerbation from those that arise as a consequence of chronic inflammation. Generalizability may also be limited by ancestry and sex. Allele frequencies and linkage disequilibrium patterns vary across populations, while sex imbalance may also affect the generalizability of findings, particularly in strongly female-predominant diseases such as SLE. Evidence supporting the prioritization of candidate regulatory mechanisms can be viewed as a continuum, from disease-associated epigenomic differences and genetic or molecular colocalization to chromatin-contact-based variant-to-gene prioritization and functional perturbation. Although these approaches progressively strengthen causal inference, none alone establishes a causal disease mechanism. Allele-specific perturbation and validation in disease-relevant primary cells or tissues can provide further support for specific variant–regulatory element–gene relationships.

Many epigenomic studies have relied on peripheral blood-derived cells or *in vitro* stimulation of T cells from healthy donors. Although these approaches are valuable for identifying cell-intrinsic regulatory mechanisms, T-cell states within inflamed tissues are shaped by local cytokines, metabolic conditions, antigen specificity and clonal expansion, and interactions with immune and stromal cells. Candidate regulatory mechanisms should therefore be validated in disease-relevant tissues or experimental systems that recapitulate these interactions and inflammatory environments, ideally integrating epigenomic profiling with T-cell receptor sequencing and surface-protein measurements. Advances in single-cell multiome profiling, single-cell three-dimensional genome analysis, and spatial epigenomics may help address this challenge by enabling characterization of regulatory states within their native cellular and tissue contexts.

Translational applications of epigenomic profiling include biomarkers for disease prediction or patient stratification, identification of candidate therapeutic targets, and direct pharmacological targeting of epigenetic regulators. Particularly for therapeutic target selection, the association of an epigenomic alteration with disease is not sufficient to establish that the implicated regulator is a safe or selective therapeutic target, as many epigenetic regulators act broadly across cell types and genomic loci and may therefore cause unintended immunosuppression or other toxicities. Future therapeutic approaches will need to achieve greater selectivity for disease-associated T-cell states or inflamed tissues. Ultimately, epigenomic analysis may provide a framework not only for linking genetic risk for autoimmune disease to specific cellular states and pathogenic functions but also for improving disease prediction, patient stratification, and therapeutic target selection.
